# Proteomic and single-cell analysis shed new light on the anti-inflammatory role of interferonβ in chronic periodontitis

**DOI:** 10.3389/fphar.2023.1232539

**Published:** 2023-10-09

**Authors:** Jieying Liu, Tianle Li, Shunhao Zhang, Eryi Lu, Wei Qiao, Huimin Chen, Peng Liu, Xiaoyue Tang, Tianfan Cheng, Hui Chen

**Affiliations:** ^1^ Department of Medical Research Center, Peking Union Medical College Hospital, Chinese Academy of Medical Sciences & Peking Union Medical College, Beijing, China; ^2^ Faculty of Dentistry, Prince Philip Dental Hospital, University of Hong Kong, Pokfulam, China; ^3^ State Key Laboratory of Oral Diseases, National Clinical Research Center for Oral Diseases, West China Hospital of Stomatology, Sichuan University, Chengdu, China; ^4^ Department of Stomatology, Renji Hospital, Shanghai Jiao Tong University School of Medicine, Shanghai, China

**Keywords:** single-cell analysis, chronic periodontitis, macrophage, IFNβ, ISG15, IL10, proteomics

## Abstract

Periodontitis, a condition that results in periodontal attachment loss and alveolar bone resorption, contributes to the global burden of oral disease. The underlying mechanism of periodontitis involves the dysbiosis and dyshomeostasis between host and oral microbes, among which the macrophage is one of the major innate immune cell players, producing interferon β (IFNβ) in response to bacterial infection. The objective of this research was to examine the interaction of macrophages with periodontitis and the role and mechanism of IFNβ on macrophages. IFNβ has been shown to have the potential to induce the differentiation of M1 to M2 macrophages, which are stimulated by low levels of lipopolysaccharide (LPS). Additionally, IFNβ has been demonstrated to promote the production of ISG15 by macrophages, which leads to the inhibition of the innate immune response. Moreover, our investigation revealed that IFNβ has the potential to augment the secretion of ISG15 and its downstream cytokine, IL10, in LPS-stimulated macrophages. Single-cell analysis was conducted on the gingival tissues of patients with periodontitis, which revealed a higher proportion of macrophages in the periodontitis-diseased tissue and increased expression of IFNβ, ISG15, and IL10. Gene Set Enrichment Analysis indicated that bacterial infection was associated with upregulation of IFNβ, ISG15, and IL10. Notably, only IL10 has been linked to immunosuppression, indicating that the IFNβ-ISG15-IL10 axis might promote an anti-inflammatory response in periodontitis through IL10 expression. It is also found that macrophage phenotype transitions in periodontitis involve the release of higher levels of IFNβ, ISG15, and IL10 by the anti-inflammatory M2 macrophage phenotype compared to the pro-inflammatory M1 phenotype and myeloid-derived suppressor cells (MDSCs). This implies that the IFNβ-induced production of IL10 might be linked to the M2 macrophage phenotype. Furthermore, cell communication analysis demonstrated that IL10 can promote fibroblast proliferation in periodontal tissues via STAT3 signaling.

## 1 Introduction

Chronic periodontitis, characterized by the formation of periodontal pocket, progressive attachment loss, and alveolar bone resorption, has been recognized as a global burden of oral diseases by the World Health Organization (WHO) (Genco et al., 2020). In dentate adults, the prevalence of periodontitis was estimated to be around 62%, with severe periodontitis affecting 23.6% of this population between 2011 and 2022 (Trindade et al., 2023). Periodontal diseases accounted for 3.5 millions of years lived with disability (YLD) globally in 2016 (GBD, 2016 Disease and Injury Incidence and Prevalence Collaborators, 2017). These statistics highlight the significant impact of chronic periodontitis on public health. Not only is periodontitis a severe oral problem that causes tooth loss and impaired mastication, but also it has a profound impact on the general health (Clark et al., 2021). Periodontal disease and other systemic chronic inflammatory diseases appear to be intertwined, as they share several features of the immunoinflammatory response and build complicated networks, which contribute to the persistent inflammation in the periodontal tissue (Clark et al., 2021). This persistent inflammation contributes to the time-consuming nature of treatment and the variability of treatment outcomes among patients (Cardoso et al., 2018). Hence, anti-inflammatory cytokine-based therapy shows promising potential for treating chronic periodontitis (Cardoso et al., 2018).

Macrophages, being the primary cellular elements of innate immunity, are crucial for tissue homeostasis. They are the major host defence against microorganisms and have the capacity to rapidly migrate to the affected site (Benoit et al., 2008). Beyond their roles in host defense, macrophages have the capacity to release both pro-inflammatory and anti-inflammatory factors, induce programmed cell death, and facilitate adaptive immunity through antigen presentation (Stutz et al., 2021; Wu et al., 2022). In recent years, several studies have focused on the subtypes and adaptability of monocytes/macrophages. Macrophages are mainly divided into two phenotypes based on their function: M1 and M2 (Stout and Suttles, 2004). During the early stage of inflammation, activated M1 macrophages release TNFα and IL12, while M2 macrophages stimulate IL10 production to play an anti-inflammatory role during the resolution phase (Almubarak et al., 2020). The dynamic balance of M1/M2 macrophages is crucial for the pathogenesis of periodontal diseases. In addition to M1 and M2, Myeloid-derived suppressor cells (MDSCs) are a type of pathologically activated monocytes or neutrophils with potent immunosuppressive activity (Veglia et al., 2021). Previous studies have reported that MDSCs can expand systemically during immune responses to various bacterial infections (Tam et al., 2014; Tsiganov et al., 2014), including periodontitis (Leija-Montoya et al., 2023). Moreover, the percentage of MDSCs has been shown to increase with accelerated aging (Flores et al., 2017), indicating the potential involvement of MDSCs in aging-related changes in immune function and disease progression. Type I interferons (IFNs), including IFNα and IFNβ, are produced by the host in response to bacterial invasion via the TLR4 signalling pathway (McNab et al., 2015). They are typically mediated by IFNAR-1 and IFNAR-2, which are type I IFN receptors, in innate immune cells such as macrophages (Ivashkiv and Donlin, 2014). IFNβ expression was elevated in patients with periodontitis (Kajita et al., 2007). Intriguingly, some studies reported that IFNβ could reduce the number of mature osteoclasts, preventing bone tissue resorption in osteoporosis model (Takayanagi et al., 2002; Takaoka and Taniguchi, 2003). However, the role and mechanism of IFNβ in immunomodulation and tissue protection have not been well-investigated in periodontitis.

In this study, inflammatory model was established by using an LPS-induced RAW 264.7 cell model. Upon stimulating macrophages with LPS, a significant increase of ISG15 was observed in the IFNβ-LPS co-stimulated group in comparison to the LPS group. The biological functions of ISG15 were also crosslinked, and the results suggested its association with anti-inflammatory functions. Moreover, IFNβ was found to significantly enhance the expression of ISG15 and its downstream cytokine, IL10, in LPS-stimulated macrophages. Additionally, single-cell sequencing (scRNA-seq) was conducted on gingival tissues obtained from both healthy individuals and patients with periodontitis to investigate differential gene expression in different cell types, the functions of differential genes, intercellular communication networks, and the origin of those differential genes.

## 2 Materials and methods

### 2.1 Cell culture

The murine macrophage RAW 264.7 cells (National Infrastructure of Cell Line Resource, China) were cultured in RPMI 1640 medium (Gibico) with 1% penicillin-streptomycin (Gibico) and 10% fetal bovine serum (Gibico) supplementation. The cells were incubated at 37°C in an air humidified incubator with 5% CO_2_ supply. Cell numbers were adjusted to 1×10^6^ cells/well in 6-well plates (Corning), and incubated with various experimental conditions: 1) 10 ng/mL IFNβ (PeproTech) for 24 h (labelled as group F); 2) Blank for 72 h (labelled as group O); 3) 100 ng/mL LPS (Sigma Aldrich) for 72 h (labelled as group L); 4) 100 ng/mL LPS for 72 h followed by the last 24 h combination incubation in 10 ng/mL IFNβ (labelled as group LF). Images of cells morphology at different time points were taken with a Zeiss Axiovert microscope (Carl Zeiss Inc., Thornwood, NY, United States).

### 2.2 Cell lysis and protein extraction

The cell were lysed and extracted using Micro Sample Preparation Kit (Omicsolution). Briefly, after removing media, the cells were washed three times with cold phosphate-buffered saline (PBS) and lysed on ice for 10min with cold lysis buffer in the kit. The lysis samples were further centrifugated at 14,000 g for 15 min at 4°C, and the supernatant was collected. The protein concentration was determined using BCA protein assay kit (Thermo Scientific, Branchburg, NJ, United States). A total of 30 μg of protein per sample was used and then prepared with the Micro Sample Preparation Kit according to the instructions. After proteolysis at 37°C for 2 h, the samples were desalted, dried, and dissolved in 20 μL of 0.1% formic acid (FA) for subsequent LC-MS/MS assays.

### 2.3 Direct DIA proteomics analysis

EASY-nLC 1200 UHPLC and Q-Exactive HF (Thermo Scientific, Rockwell, IL, United States of America) were used to perform the LC-MS/MS analysis. After loading all the samples onto the trap column, the eluent was further passed through a reversed-phase analytical column (75 mm × 500 mm, 2 mm, MONOTECH). A gradient of 5%–30% buffer B was used for elution (flow rate = 600 nL/min; 0.1% FA in 99.9% acetonitrile) over 60 min. The MS parameters were set up as follows: A resolution of 120,000 and a range of 350–1,250 m/z were used for the full scan; the AGC was 3e^6^; and the injection time was under 50 m. The MS/MS parameters were set up as follows: resolution was set up as 30000, the AGC target was 1e^6^ and the auto maximum injection time was used.

Spectronaut Pulsar X software (Biognosys, AG, Schlieren, Switzerland) was used to search raw data from DIA proteomics analysis with the default settings. Based on the iRT calibration strategy, an optimal window for XIC extraction was identified. All proteins were identified based on two unique peptides following the Q-value filter.

### 2.4 Bioinformatics analysis of proteomics

The *p*-value was set to 0.05 for protein identifications. Gene Ontology (GO) enrichment pathway analyses of the significantly different proteins between LF and F groups were performed using clusterProfiler R package (Wu et al., 2021). Protein interactions were analysed using STRING database (https://string-db.org), and the interactions with a combined score greater than 0.4 were chosen to build the PPI networks by Cytoscape software.

### 2.5 Data acquisition and cells grouping

The scRNA-seq data of healthy people and periodontitis patients (GSE164241) were downloaded from the Gene Expression Omnibus (GEO). After the removal of the batch effects (Stuart et al., 2019), data were integrated from 16 separate samples (GSM5005049, GSM5005050, GSM5005051, GSM5005052, GSM5177039, GSM5005056, GSM5005057, GSM5177040, GSM5005058, GSM5005059, GSM5005060, GSM5005061, GSM5005062, GSM5177042, GSM5177043 and GSM5177044). Quality control was performed by using Seurat (version 3.1.5) (Qian et al., 2021) and Harmony (Korsunsky et al., 2019) of R package. After that, 63,159 cells were remained and used for the subsequent study. The top 2000 variable genes were selected using the FindVariableGenes function of the Seurat package. Subsequently, the Principal Component Analysis (PCA) and Uniform Manifold Approximation and Projection (UMAP) were executed. Several cell types were identified by marker analysis. Cell type abundance analysis revealed the proportions of cell clusters in the periodontitis group versus the healthy group, and volcano plots depicted the up- and downregulation of cells in the periodontitis group.

### 2.6 Differential gene analysis and enrichment analysis

The differential genes between various clusters were determined by using FindMarkers function of Seurat package. The significance levels were chosen with the criteria of an adjusted *p*-value <0.05 and a log2-fold change >0.5. The biological features of genes in the periodontitis group were investigated using Gene Set Enrichment Analysis (GSEA) and calculated with Normalized Enrichment Score (NES) based on the Gene Ontology (GO) data set via the GseaVis package (Junjunlab, 2022). The threshold will be established at a *p*-value <0.05.

### 2.7 Pseudotime analysis

The Monocle3.0.2 Bioconductor package (Trapnell et al., 2014) was used to perform pseudotime analysis of macrophage Trajectories. The Seurat object previously generated from the macrophages subcluster was imported into Monocle3 and run on the normalized counts matrix for the subclustered macrophage dataset. UMAP dimensional reduction and cell clustering were performed using the “cluster_cells” function to plot a principal graph through the UMAP coordinates. The “learn_graph” function was used to plot this principal graph. The most significantly expressed genes with the greatest q-values were plotted on a heatmap of expression over pseudotime using the “plot_pseudotime_heatmap” function in Monocle.

### 2.8 Identification of cell-cell interactions in periodontitis

Cell-cell interactions was performed by using the CellChat (Jin et al., 2021) and cellcall (Zhang et al., 2021). After calculating and examining cell-cell communication in periodontitis, a number of crucial ligand-receptor interactions between different cells were selected.

### 2.9 Quantitative real-time PCR (qPCR)

The RAW264.7 cells were collected and the total RNA was extracted using RNA Isolation Kit (Beyotime). First Strand cDNA Synthesis Kit (Beyotime) was utilized to generate cDNA. The amplification and detection of cDNA targets were performed using SYBR Green Premix Ex Taq (Takara) on a StepOne Plus Real-time PCR system (Applied Biosystems). GAPDH was used as the internal control. The relative difference was calculated using the 2^(-ΔΔCT) method and expressed as fold change.

### 2.10 Western blotting

The Western blot assays conducted in this study followed previous protocols (W et al., 2021). Briefly, RAW264.7 cells were lysed with RIPA lysis buffer, and the BCA Protein Assay Kit was used to measure the protein concentration. The proteins were separated by SDS-PAGE and transferred onto a nitrocellulose filter membrane before being incubated with antibodies, including anti-GAPDH, anti-ISG15 (CST #2743) and anti-IL10 (Abcam, ab189392). The specific bands were detected using ECL reagent (Tanon) on radiograph films. The developed films were then scanned and quantified using Quantity One software (Tanon5200). The expression levels of the targeted proteins were quantified relative to GAPDH in the same sample, and these levels were normalized to the respective control group, which was arbitrarily set at one-fold.

### 2.11 Statistical analysis

Statistical analyses were performed with Prism GraphPad version 9.1.1 software. All data were expressed as means ± standard deviation (SD). To analyze the differences, we utilized one-way ANOVA followed by Tukey’s *post hoc* multiple comparison test and Kruskal–Wallis test. The selected level of significance was a value of *p* < 0.05.

## 3 Results

### 3.1 Macrophage morphology

**FIGURE 1:**
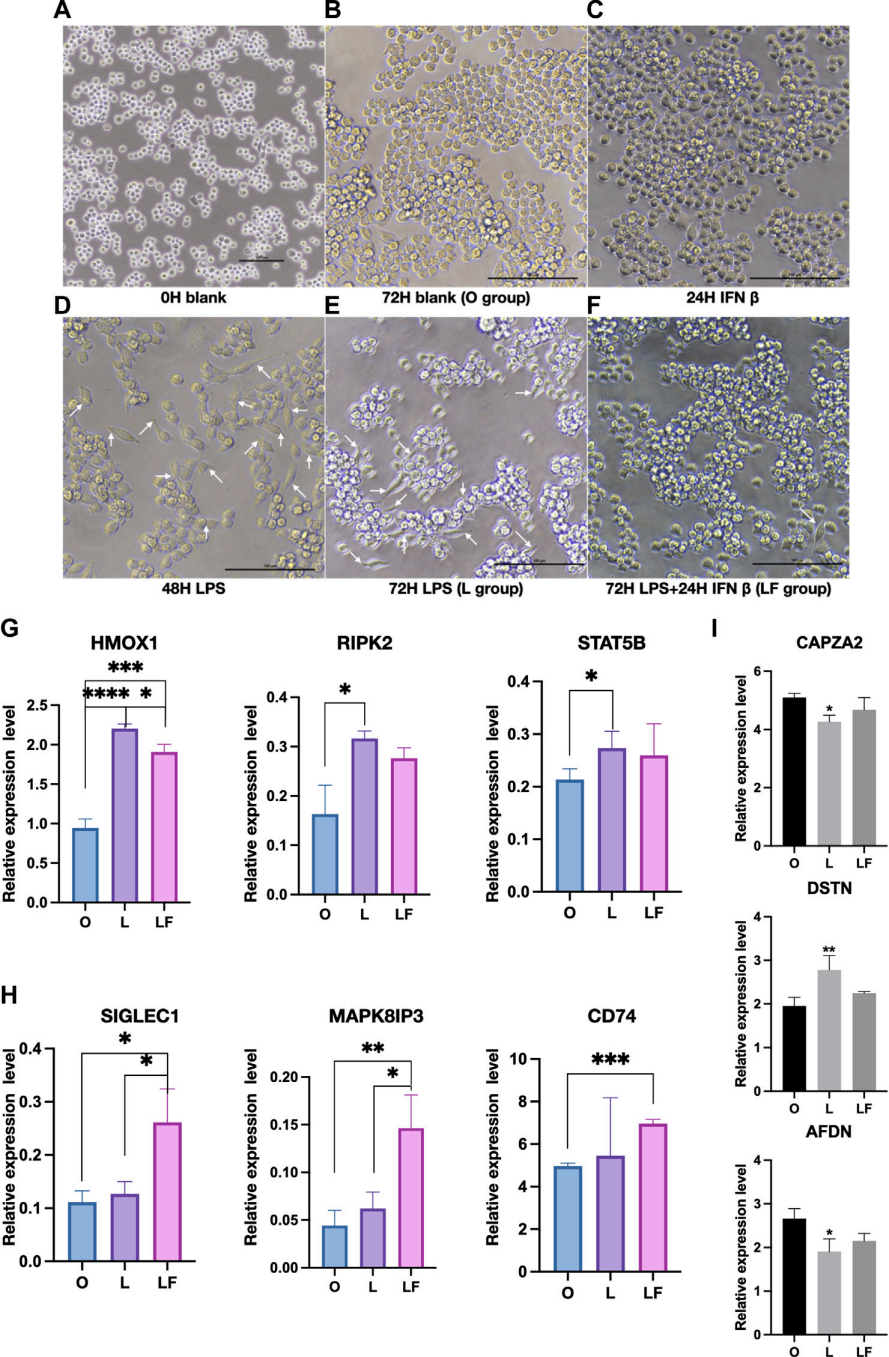
Macrophages morphology and protein expression in different groups (p < 0.05). **(A–F)** Images was detected by microscope in F, L and LF group. (White arrow: spindle-shaped cells). **(G)** Proteins expression of M1 markers in the O, L, and LF groups. **(H)** Proteins expression of M2 markers in the O, L, and LF groups. **(I)** The expression of cytoskeletal proteins in different groups (compared to O group).

In this study, a non-stimulated group was employed as the control to evaluate the effects of different treatments on macrophage morphology. Notably, macrophages in the non-stimulated group displayed a rounded shape, which is considered as a normal cellular morphology (Figure 1A) (Ning et al., 2018). The 72H blank group and IFNβ-induced group (F group) maintained the rounded shape as control group (Figures 1B, C). The macrophages were simulated with LPS to mimic an inflammatory state (L group). After 48 and 72 h of stimulation with LPS, RAW 264.7 cells were found to demonstrate spindle-shaped which has been reported to be characteristic of M1 macrophages (Figures 1D, E) (Ning et al., 2018). After 48 h of LPS stimulation, IFNβ and LPS were further co-treated for 24 h to evaluate the effect of IFNβ on macrophages following LPS stimulation (LF group). Remarkably, the addition of IFNβ at 48 h appeared to reverse the spindle-shaped morphology and promote a round-shaped morphology, which are characteristic of M2 macrophages (Figure 1F) (Ning et al., 2018). To further assess the changes in macrophage polarization, the protein expression of M1 markers (HMOX1, RIPK2 and STAT5B) and M2 markers (SIGLEC1, MAPK8IP3 and CD74) were evaluated. The results indicated that M1 markers (RIPK2 and STAT5B) were significantly expressed in the L group (Figure 1G), whereas M2 markers (SIGLEC1, MAPK8IP3 and CD74) were significantly expressed in the LF group (Figure 1H). Although HMOX1 expression was significantly increased in both the L and LF groups compared to the control group, HMOX1 expression was significantly higher in the L group compared to the LF group (Figure 1G). These results suggest that LPS might activate macrophages to differentiate into an M1 phenotype, but IFNβ could reverse such differentiation. To investigate the potential mechanism underlying the effects of IFNβ on reversing macrophage morphology induced by LPS, we examined changes in cytoskeletal proteins, including Capping Actin Protein Of Muscle Z-Line Subunit Alpha 2 (CAPZA2), Actin Depolymerizing Factor (DSTN), and Adherens Junction Formation Factor (AFDN) (Figure 1I). Our results showed that these cytoskeletal proteins were significantly altered in the L group but not in the LF group, suggesting that they could be changed after LPS stimulation but could be reversed by IFNβ treatment, which was consistent with previous study (Vergara et al., 2010). Therefore, our results suggest that LPS stimulation can activate M1 macrophages, which may be associated with an inflammatory state, while IFNβ can effectively reverse LPS-induced changes in macrophage morphology by regulating the cell cytoskeleton and promote M2 macrophage polarization to achieve an anti-inflammatory state (Bertani et al., 2017).

### 3.2 Differentially expressed proteins and gene ontology (GO) analysis

**FIGURE 2:**
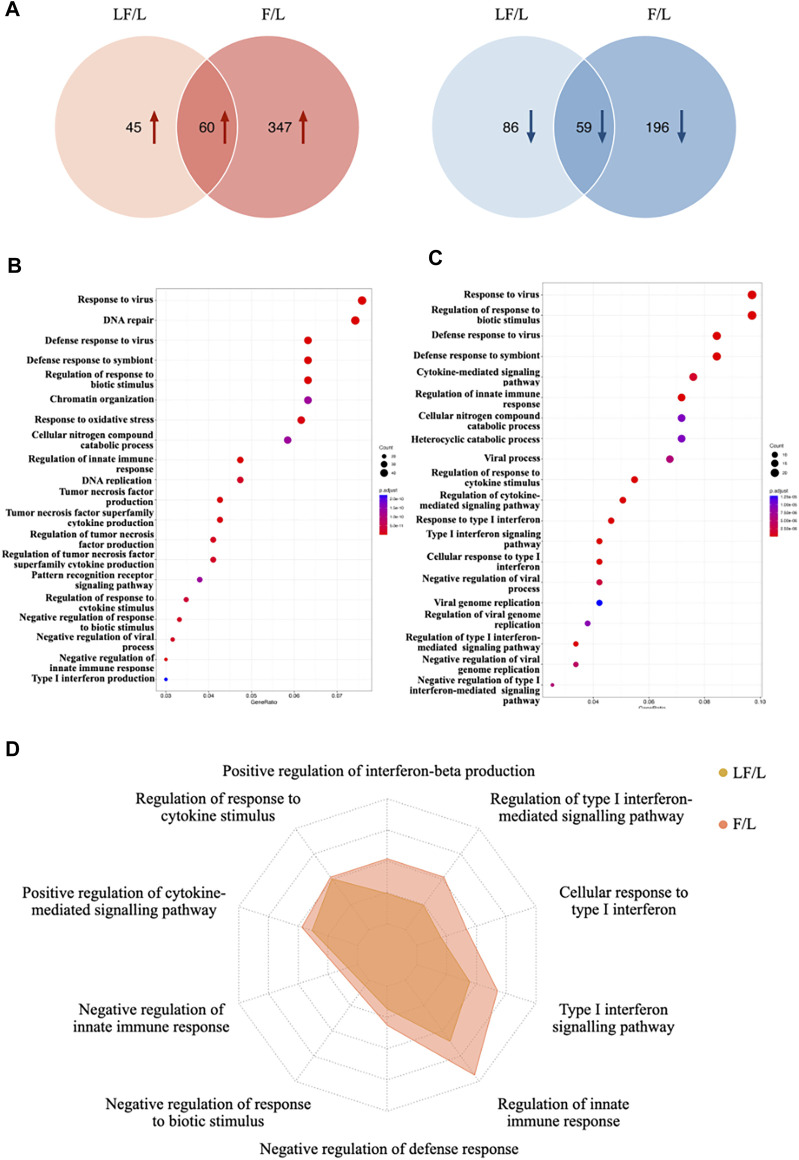
Differential proteins expression in LF and F groups (compared to L group). **(A)** Venn diagram of differentially expressed proteins between LF and F groups. **(B)** Gene oncology enrichment analysis in F group. **(C)** Gene oncology enrichment analysis in LF group. **(D)** GeneRatio distribution in target GO terms for F and LF groups.

To investigate the role of IFNβ in the context of inflammation, a comparison was made between the LF group and the L group, as well as between the F group and the L group. Differentially expressed proteins were then identified (Supplementary Table S1). In specific, a total of 250 proteins were significantly altered in the LF group compared to the L group, with 105 upregulated proteins and 145 downregulated proteins. In comparison to the L group, the F group contained 662 significantly changed proteins, 407 of which were upregulated and 255 of which were downregulated. Between the LF and F groups, there were 60 upregulated and 59 downregulated proteins (Figure 2A). In order to identify the activated biological functions, gene ontology analysis was performed based on the differentially expressed proteins (Figures 2B,C). Ten representative GO terms related to immunity were selected. The GeneRatio between the L and LF groups were compared. Our results showed that the LF group exhibited a higher GeneRatio in several key biological functions, including four terms related to the regulation of type I interferon (cellular response to type I interferon, type I interferon signaling pathway, regulation of type I interferon-mediated signaling pathway, positive regulation of interferon-beta production), four terms related to innate immune defense (regulation of innate immune response, negative regulation of defense response, negative regulation of response to biotic stimulus, and negative regulation of innate immune response), and two terms related to cytokine-mediated regulation (regulation of response to cytokine stimulus and positive regulation of cytokine-mediated signaling pathway) (Figure 2D). Overall, our findings suggested that IFNβ may play a critical role in responding to bacterial infection and regulating anti-inflammatory processes in LPS-induced inflammatory macrophages, compared to those in the non-treated macrophages. Thus, IFNβ could prevent the inflammatory response and prevent tissue destruction when infections occur.

### 3.3 Relationship between differential genes and biological functions

**FIGURE 3:**
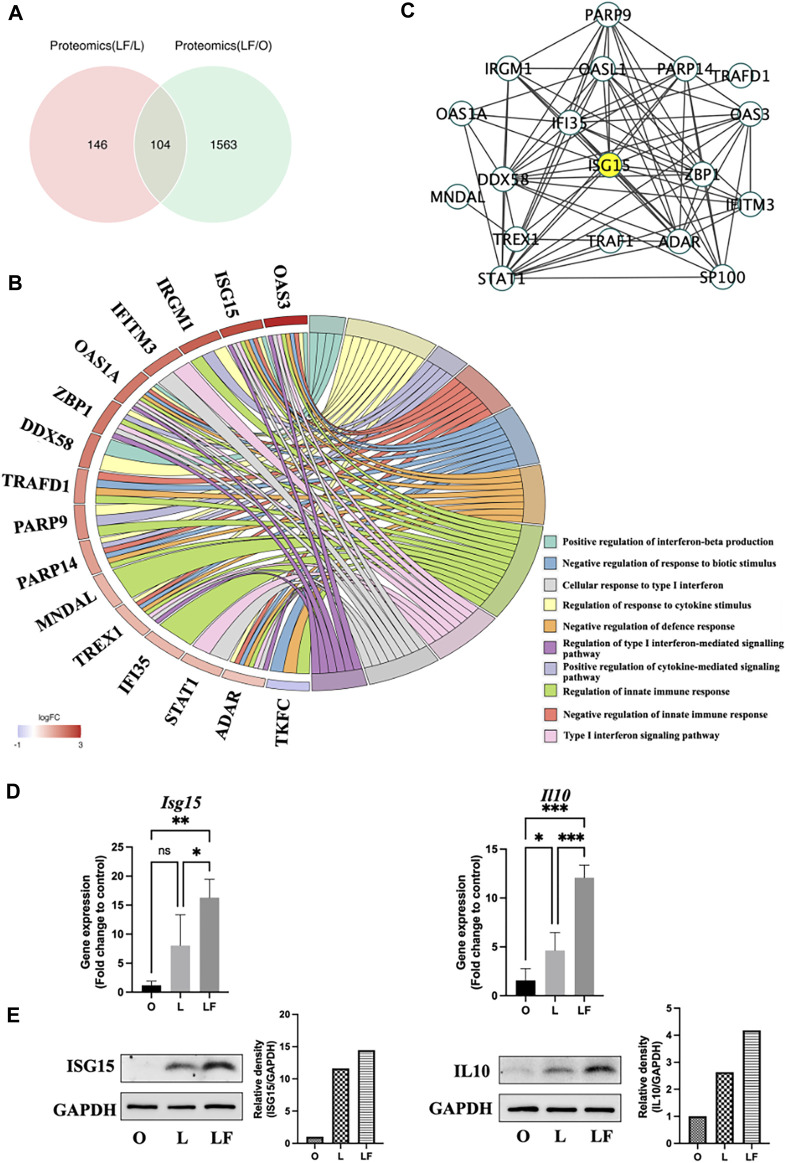
Common differential proteins expression between LF/L and LF/O (p < 0.05). **(A)** Venn diagram of differentially expressed proteins between LF/L and LF/O. **(B)** Association of differential proteins with target GO terms. **(C)** PPI network of differential proteins linked to target GO terms. **(D)** qPCR of *Isg15* and *Il10* gene expression in O, L, and LF groups. **(E)** Western blot analysis of ISG15 and IL10 protein expression in O, L, and LF groups.

A co-analysis of the differential protein expression was performed between the LF-compared-L group (LF/L) and the LF-compared-O group (LF/O). The proteomic analysis revealed significant alterations in a total of 250 proteins in the LF/L group (*p* < 0.05), and 1,667 differential proteins in the LF/O group (*p* < 0.05). Particularly, there were 104 common differentially expressed proteins in both LF/L and LF/O groups (Figure 3A). Within the set of 104 common differentially expressed proteins, 19 proteins were found to be related to the biological functions, which was consistent with our findings of GO analysis. Notably, 18 of these proteins were found to be upregulated, while one was downregulated (Figure 3B). In particularly, OAS3 and ISG15 were highest expressed and correlated with the greatest number of target biological functions. To investigate the relationships between these 19 proteins, protein-protein interaction analysis was conducted. The results reveal that ISG15 interacts with 14 other proteins. As a result, ISG15 is suggested to be the most central protein within the protein network involved in specific biological functions (Figure 3C), Our finding is consistent with other evidence that has shown ISG15 could induce IL10 in response to IFNβ stimulation (Swaim et al., 2019).

### 3.4 The expression of ISG15 and IL10 in different groups

The results of qPCR revealed a significantly higher expression of *Isg15* in LF group compared to L (*p* < 0.05) and O groups (*p* < 0.01). Nonetheless, there was no significant difference observed in the expression of *Isg15* between L and O groups. Similarly, the gene expression of *Il10* was also highest in LF group (*p* < 0.001) compared to O and L groups. In addition, the data indicated a minor increase in the expression of Il10 in the L group relative to O group (*p* < 0.05) (Figure 3D). The Western blot analysis revealed a slight increase in the expression of ISG15 and IL10 in the L group compared to O group, presumably attributed to the activation of both pro- and anti-inflammatory responses triggered by LPS. It should be noted that the LF group secreted the highest amounts of ISG15 and IL10, indicating that IFNβ further amplified the expression of these cytokines in LPS-induced inflammatory model (Figure 3E).

### 3.5 Identification of main cell types in periodontitis tissue

**FIGURE 4:**
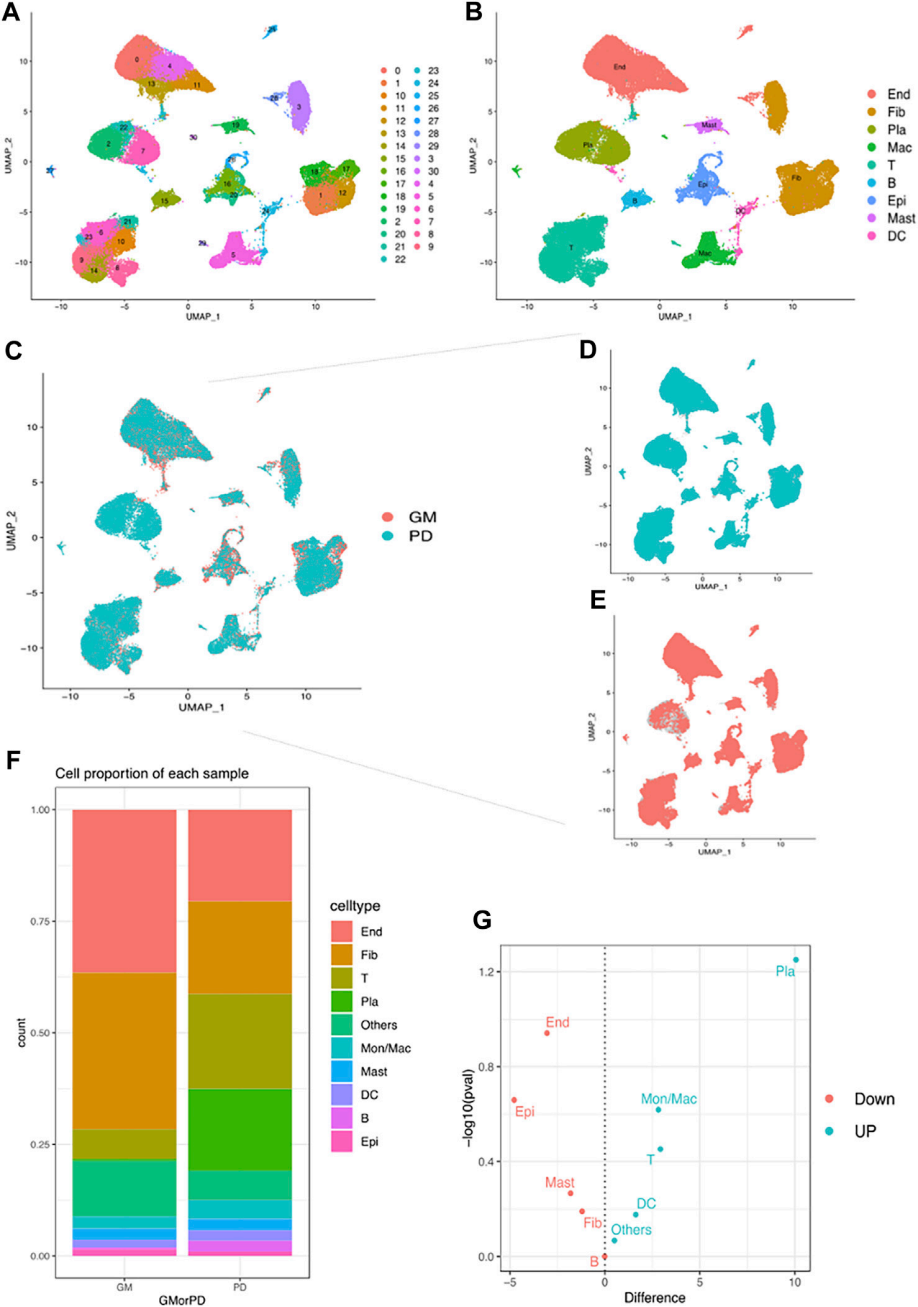
Single-cell analysis demonstrates different cell clusters. **(A)** UMAP plot of the single-cell profile coloured by cluster. **(B)** UMAP plot of the single-cell profile coloured by major cell types: Fib, End, Pla, T, Mon/Mac, Epi, B, Mast and DC. **(C–E)** UMAP plot of cell clusters coloured by GM and PD. **(F)** Proportions of different cell types in GM and PD. **(G)** volcano plot showed the up/down expression cells in PD.

The scRNA-seq data GSE164241 (Williams et al., 2021) downloaded from the GEO database was used to comprehensively classify the cells present in gingival mucosa of healthy people (GM) and periodontitis patients (PD). After quality control (Supplementary Figures S1, S2, S3), 63159 cells were divided into 30 clusters (Figure 4A). Subsequently, ten major cell types were identified using maker genes (Figure 4B), which included fibroblasts (Fib), endothelial cells (End), plasma cells (Pla), T cells T), monocytes/macrophages (Mon/Mac), epithelial cells (Epi), B cells B), mast cells (Mast), and dendritic cells (DC). A bar chart presenting the proportion of stromal cells and immune cells indicated that stromal cells (Fib, End, Epi, and Mast) were the major cell types seen in the healthy group, while immune cells (Pla, T, Mon/Mac, B, and DC) played a crucial role in PD (Figure 4C). Further analysis revealed that the abundance of stromal cells was substantially reduced in PD, whereas the number of immune cells, particularly plasma cells and macrophages, was substantially increased (Figure 4D). These findings suggest that plasma cells and macrophages may potentially play pivotal roles in the development of chronic periodontitis.

### 3.6 Genes associated with IFNβ - IL10 network were highly expressed in periodontitis

**FIGURE 5:**
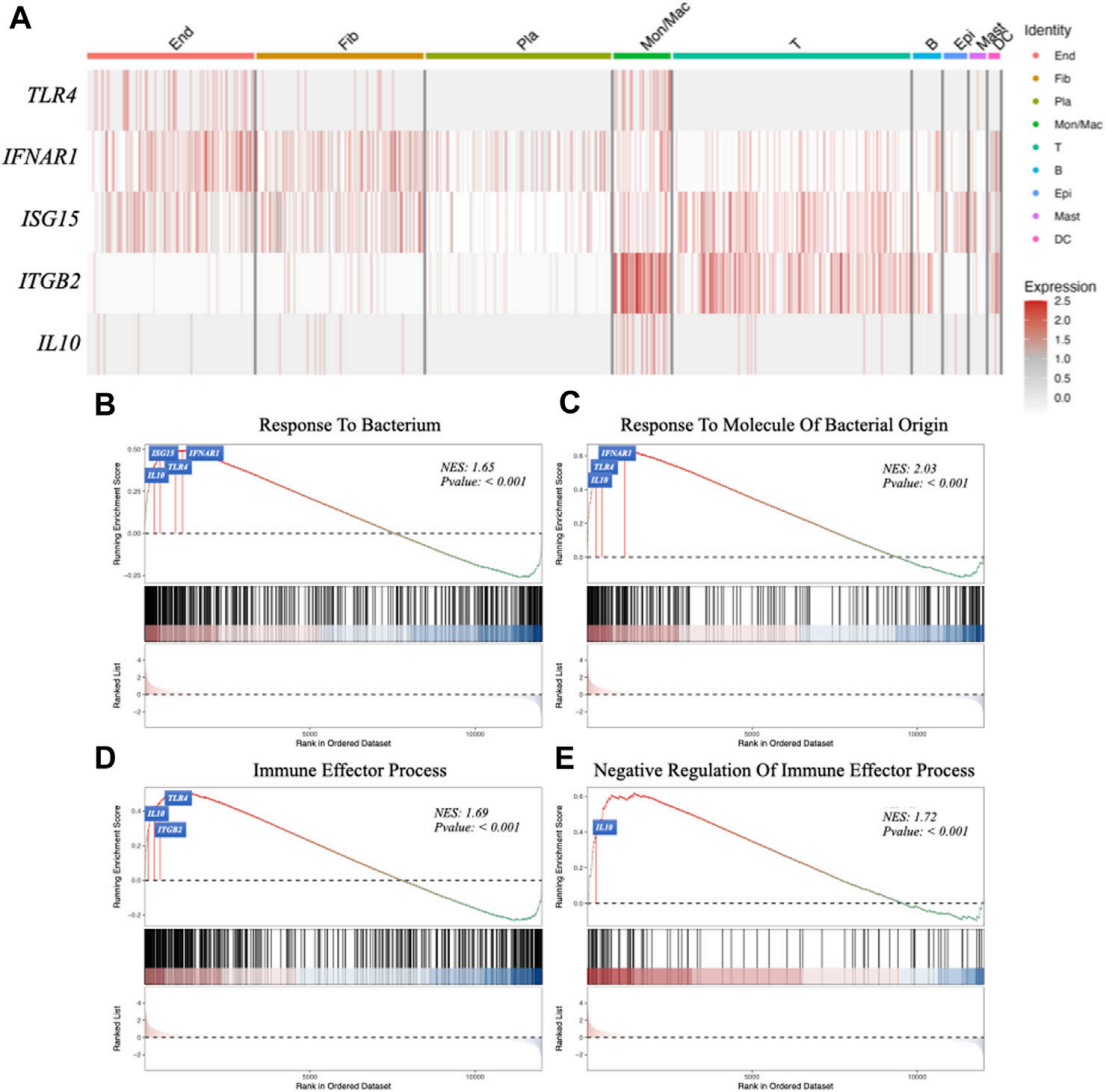
Gene expression analysis in different cell clusters and Gene Set Enrichment Analysis (GSEA). **(A)** The expression of target genes (*TLR4*, *IFNAR1*, *ISG15*, *ITGB2* and *IL10*) in different cell clusters. **(B–E)** GSEA using GO data set: response to bacterium, response to molecule of bacterial origin, immune effector process, negative regulation of immune effector process.

In the periodontitis group, macrophages were observed to exhibit elevated expressions of *TLR4*, *IFNAR1* (the receptor of IFNβ), *ISG15*, *ITGB2* (the receptor of ISG15), *IL10* (Figure 5A). This indicates that macrophages might be involved in regulating periodontitis via the IFNβ–IL10 cytokine network. In addition, Gene Set Enrichment Analysis (GSEA) was performed to explore the macrophage cluster in patients with periodontitis. Four Gene Ontology (GO) terms were found to be enriched in two bacterial infection response and two immune processes (Figures 5B–E): *TLR4*, *IFNAR1*, *ISG15*, and *IL10* were associated with “response to bacterium” (GO:0009618) (Figure 5C), *IFNAR1*, *TLR4*, and *IL10* were related to “response to bacterial molecule” (GO:0002237) (Figure 5D), *TLR4*, *IL10*, and *ITGB2* were associated with “immune effector process” (GO:0002252) (Figure 5E). Furthermore, *IL10* was linked to “negative regulation of immune effector” (GO:0002698) (Figure 5F). These findings suggest that macrophages may regulate the respond to bacterial components and modulate immune process in periodontitis via IFNβ-IL10 cytokine network.

### 3.7 Trajectories and functions of macrophages in periodontitis

**FIGURE 6:**
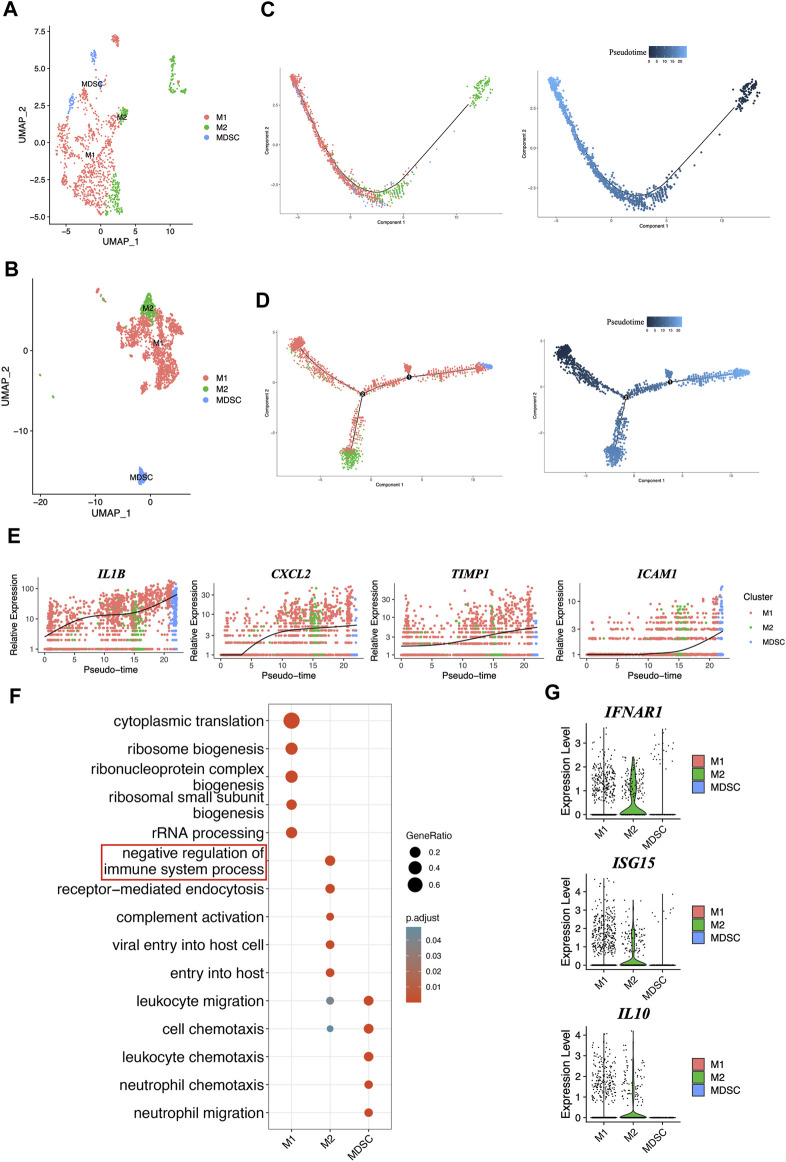
Comprehensive characterization and pseudotime analysis of macrophage phenotypes and gene expression dynamics. **(A)** UMAP plot of macrophages colored by cell type in GM. **(B)** UMAP plot of macrophages colored by cell type in PD. **(C)** Single-cell trajectories across three distinct phenotypes of macrophages and pseudotime in GM. **(D)** Single-cell trajectories across three distinct phenotypes of macrophages and pseudotime in PD. **(E)** The expression dynamics of senescent marker genes. **(F)** Gene oncology enrichment analysis in different phenotypes of macrophages. **(G)** The genes expression levels from IFNβ-IL10 network were plotted on violent plots.

Macrophages were identified for three phenotypes, M1, M2, and MDSC, in healthy people and periodontitis patients (Figures 6A, B). MDSCs were characterized by the lowest levels of *HLA-DRA* along with the highest levels of *S100A8*, *S100A9*, *S100A12* and *CXCL8* (Umansky et al., 2016; Voskamp et al., 2023) (Supplementary Figure S4). Our study found that the periodontitis group had three trajectories, M1, M2, and MDSC, while the healthy group only had two trajectories (Figures 6C, D). This might indicate that the immune response in periodontitis patients is more complex and involves a wider range of macrophage subtypes, particularly MDSCs, compared to healthy individuals. Moreover, our findings suggested that the phenotype conversion of macrophages in periodontitis patients predominantly exhibits the M1 phenotype at the beginning, with some M1 transitioning to M2, resulting in a dynamic shift between the two phenotypes. As the disease progresses, there is a gradual increase in the percentage of MDSCs, which eventually become the dominant cell type. Furthermore, the genes related to senescence (*IL1B, CXCL2, TIMP1, ICAM1*) (Yue et al., 2022) were also gradually increased, which was consistent with MDSC enhancement (Figure 6E). These findings suggested the macrophage phenotype conversion and complex interplay between aging and macrophage polarization in periodontitis.

The Gene oncology enrichment analysis showed the biological functions between M1, M2 and MDSC (Figure 6F), especially M2, it mainly participates in the negative regulation of the immune system process, and that genes involved in IFNβ-IL10 network, such as *IFNAR1*, *ISG15*, and *IL10* (Figure 6G), might help M2 regulate this process. These findings provide important insights into the function of different phenotypes of macrophages in periodontitis.

### 3.8 Identification of cell-cell interactions in periodontitis

**FIGURE 7:**
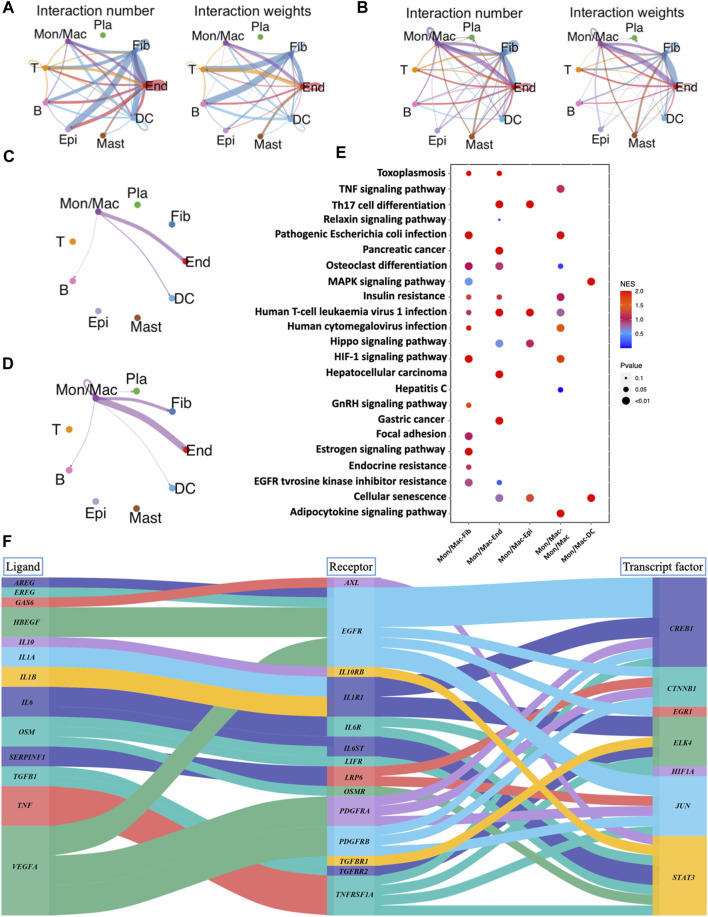
Analysis of cell-cell interactions and corresponding pathway activity. **(A)** Cell-cell interactions are illustrated by CellChat in GM. **(B)** Cell-cell interactions are illustrated by CellChat in PD. **(C)** Network diagram of the cell-cell interactions of macrophages in GM. **(D)** Network diagram of the cell-cell interactions of macrophages in PD. **(E)** Pathway activity analysis of ligand-receptor interactions between different cells. **(F)** Sankey plot of ligand-receptor-transcript factor axis for the communications between different cells.

Cell-cell interaction analysis for GM and PD revealed 9 cell types that communicate via ligand-receptor interactions (Figures 7A, B). Gingival fibroblasts, which constitute the majority of resident cells in the gingival connective tissue, contribute to periodontal health by aiding tissue repair and regeneration (Naruishi and Nagata, 2018). There was no interaction observed between fibroblasts and macrophages in GM (Figure 7C), whereas in PD, crosstalk between these 2 cell types was evident (Figure 7D). This implies that macrophages may potentially regulate fibroblasts, ultimately contributing to the development of the disease. In pathway activity analysis, pathogenic *escherichia coli* infection, osteoclast differentiation, HIF-1 signaling pathway, focal adhesion, estrogen signaling pathway and EGFR tyrosine kinase inhibitor resistance were significantly expressed in the macrophage-fibroblast crosstalk of PD (Figure 7E). It is noteworthy that focal adhesion and estrogen signaling pathways have been observed to promote fibroblast proliferation and collagen synthesis, thereby promoting extracellular matrix synthesis and re-establishing lost tissues (Wang et al., 2001; Luo et al., 2014; Koch et al., 2020). To further explore the role of *IL10* in macrophage-fibroblast crosstalk, the ligand-receptor-transcription factor axis among macrophages and fibroblasts was performed (Figure 7F). The results show that *IL10* initiates the activation of the *STAT3* transcription factors in fibroblasts, which have been previously implicated in pro-fibrotic pathways, ultimately leading to an increase in fibroblast proliferation and migration (Chakraborty et al., 2017).

## 4 Discussion

Periodontitis is a multifactorial inflammatory disease that results in loss of tooth-supporting tissues and a systemic inflammatory response. The interaction between microbial variables and the human immune system determines the severity and extent of periodontitis (Pihlstrom et al., 2005), with monocytes and macrophages playing a significant role (Cekici et al., 2014). The activation of surface receptors, especially toll-like receptors that recognize and bind surface molecules of bacteria (Cekici et al., 2014), initiates the production of pro- and anti-inflammatory cytokines (Choi et al., 2022). IFNβ has been reported to be induced by outer membrane vesicles of *Porphyromonas gingivalis*, *a* bacterium closely associated with periodontitis (Fleetwood et al., 2017). In this study, IFNβ was found to further stimulated IFNβ-mediated pathways and subsequent cytokine secretion, leading to more potent immunosuppressive effects in the LPS-induced inflammatory group when compared to the group treated with IFNβ alone. These findings suggest that the immunosuppressive effects of IFNβ might be achieved under conditions of LPS-induced inflammation.

Furthermore, the LF/L and LF/O proteomics data sets were merged to identify common differential proteins, resulting in the identification of 104 proteins associated with IFNβ-mediated and immune-related Gene Ontology (GO) phenotypes in the LF group. Importantly, ISG15 was discovered to be a highly expressed protein and a central component of the protein-protein interaction (PPI) network. As a ubiquitin-like protein induced by type I interferon signaling, ISG15 is known to stimulate downstream inflammatory cytokines, such as IL10 (Swaim et al., 2019). In an LPS-induced inflammatory model, treatment with IFNβ leads to a significant increase in the gene and protein expression of both ISG15 and IL10, suggesting that IFNβ may further enhance the secretion of these cytokines. Notably, both ISG15 and IL10 play a crucial role in the innate immune response to viral or microbial infections (Penaloza et al., 2015; Swaim et al., 2019), and the ISG15/IL-10 axis has been shown to exert an anti-inflammatory effect in bacterial infections as well (Dos Santos et al., 2018).

Single-cell analysis was conducted to investigate the differences in human gingival tissues between healthy individuals and those with periodontitis. The proportion of stromal cells were significantly reduced, while plasma and macrophages were significantly increased in the PD. Further analysis focused on the PD group and revealed the upregulation of TLR4, IFNAR1, IL10, ISG15 and its receptor ITGB2 in macrophage. Moreover, GSEA identified IFNAR1, ISG15 and IL10 as potential regulators of both bacterial infection and immune response. Of these, only IL10 exhibited association with immunosuppressive effects, indicating that the IFNβ-IL10 network is likely involved in regulating immune response to bacterial infection, while IL10 production is responsible for the immunosuppressive effect of the IFNβ-IL10 network.

To investigate the relationship between disease progression and macrophage phenotype transitions in periodontitis, our study revealed that macrophages initially reach a dynamic balance between M1 and M2 phenotypes. However, as the disease progresses, the percentage of MDSCs gradually increases, ultimately leading to their dominance. This shift in macrophage polarization was accompanied by changes in the expression of senescent cytokines. Furthermore, our functional analysis revealed that M2 macrophages are involved in regulating the anti-inflammatory process, and that genes related to the IFNβ-IL10 network, including IFNAR1, ISG15, and IL10, were mainly expressed in M2 macrophages. These findings suggest that M2 macrophages may regulate immunosuppressive processes through the IFNβ-IL10 pathway.

Gingival fibroblasts are essential for maintaining the physiological balance of connective tissue surrounding teeth, ensuring proper control of periodontitis and tissue regeneration (Scheres et al., 2011). To examine the communication between macrophages and fibroblasts, a cell-cell interaction analysis was conducted. The PD group showed evidence of interaction between macrophages and fibroblasts, while the GM group did not. KEGG enrichment analysis revealed that macrophages could regulate fibroblast proliferation through the focal adhesion and estrogen signaling pathways, contributing to the rebuilding of periodontal tissue (Wang et al., 2001; Luo et al., 2014; Koch et al., 2020). A ligand-receptor-transcription factor axis was then examined to elucidate the mechanism by which macrophage-induced IL10 regulates fibroblasts. It was found that IL10 produced by macrophages could activate STAT3 transcription factor of fibroblasts. Several studies have shown that STAT3 transcription factor plays a protective and pro-fibrosis role in fibroblasts (Kasembeli et al., 2018), suggesting that macrophages may promote fibroblast proliferation and protection via induction of IL10 and STAT3.

Despite the valuable insights gained from this study, there were some limitations that should be considered. The scRNA-seq data analyzed in this study were obtained from human periodontal tissue, while the *in vitro* model used to validate the findings was based on murine RAW 264.7 cells. Although RAW 264.7 cells were a well-characterized cell line and a valuable model for studying macrophages in periodontitis (Wu et al., 2022), there might be some differences in the responses of cells from different species. These differences could potentially influence the conclusions drawn from this study. Additionally, although our study utilized individual datasets to unveil the intricate interactions between cells and cytokines, we did not perform direct biological characterization assays, which might cause certain constraints to our findings. Specifically, under pathological conditions, some functionally important genes might be expressed, yet their associated biological characterizations might not have been clearly presented. Therefore, to further illuminate the underlying mechanisms of macrophage-mediated immune responses in periodontitis, future research should validate these findings across a broader array of experimental animal models and human subjects. By recognizing these limitations, we aim to stimulate a deeper understanding of the subject matter and foster further research in this crucial field.

In summary, our study found that IFNβ can inhibit the progression of chronic periodontitis and protect periodontal tissues via the induction of IL10 production through ISG15. Such process may be linked to macrophage M2 differentiation. The findings of this study provide insight into the immunosuppressive mechanism of IFNβ in chronic periodontitis, enhancing our understanding of the secretion anti-inflammatory cytokine in this condition. The results offer potential direction for future treatments aimed at managing chronic periodontitis and promoting the repair of periodontal tissue.

## Data Availability

The datasets presented in this study can be found in online repositories. The names of the repository/repositories and accession number(s) can be found in the article/Supplementary Material.
