# Supplementary figures and images for: Proteomic and single-cell analysis shed new light on the anti-inflammatory role of interferonβ in chronic periodontitis
Source: Front Pharmacol. 2023 Oct 9;14:1232539. doi: 10.3389/fphar.2023.1232539 (PMC10590904; doi:10.3389/fphar.2023.1232539)

**nFeature\_RNA**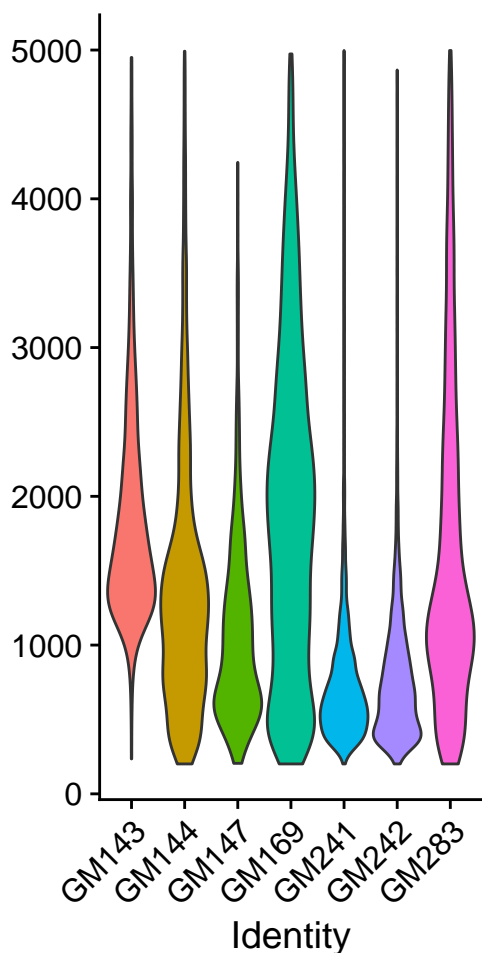**nCount\_RNA**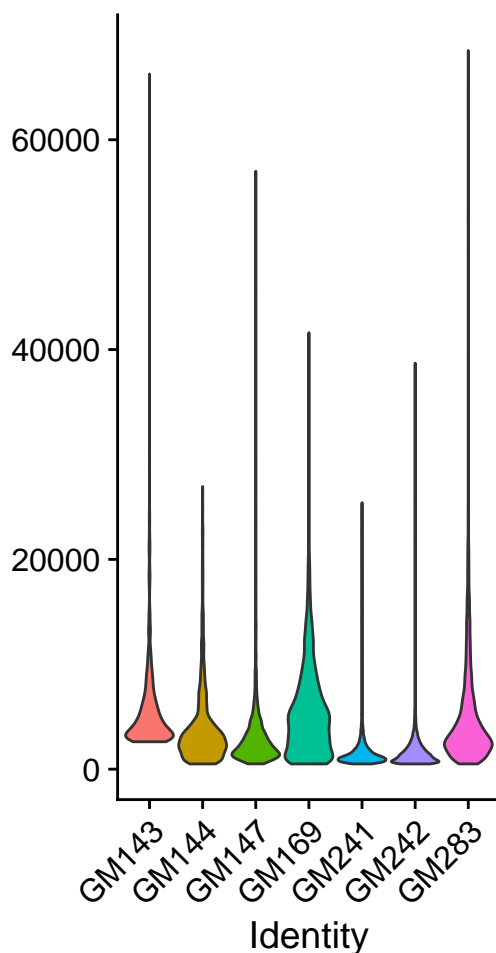**percent.mt**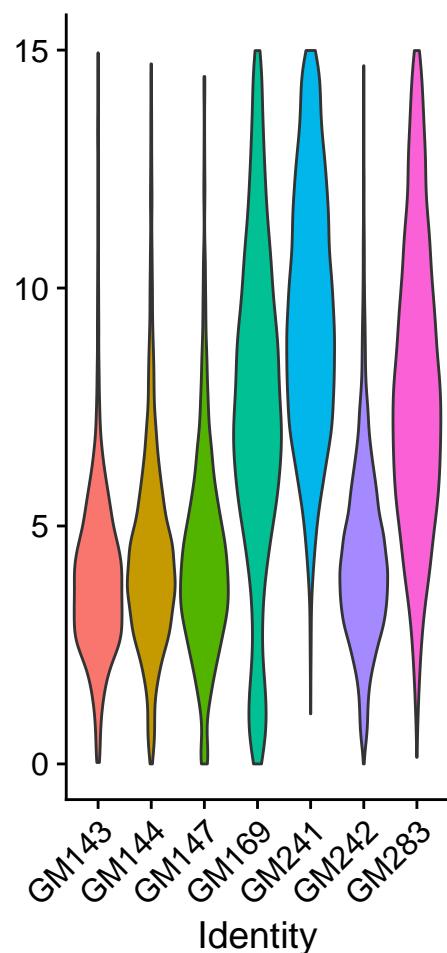**nFeature\_RNA**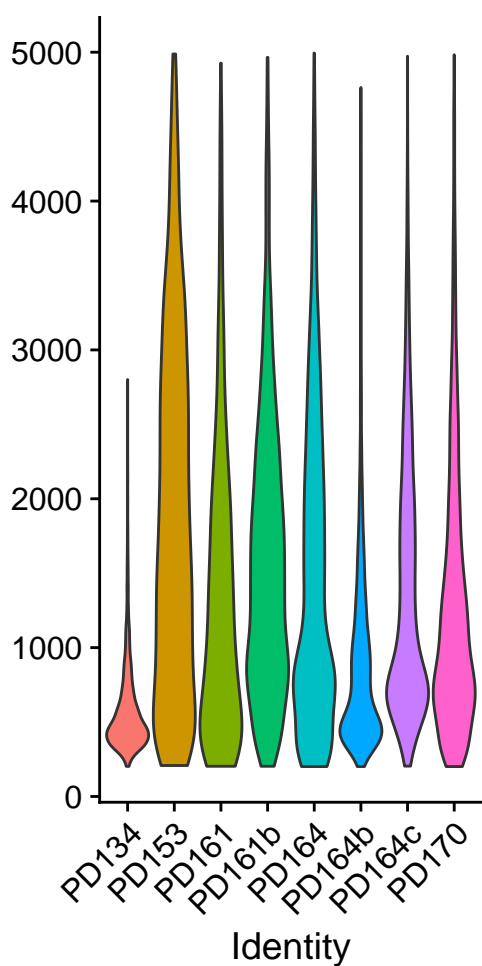**nCount\_RNA**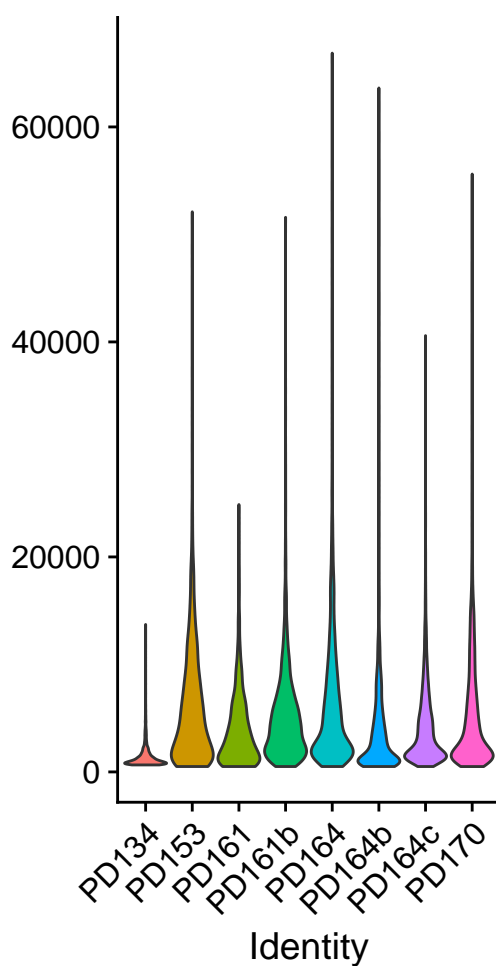**percent.mt**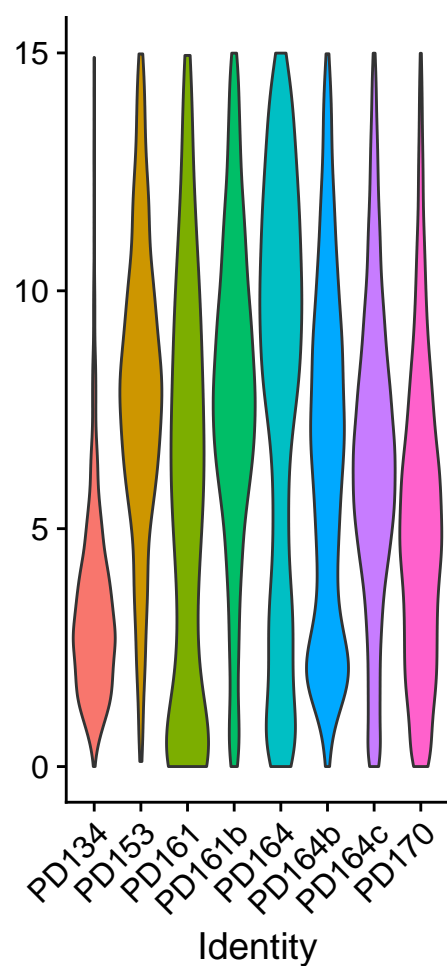

Supplement: Supplementary file 1 [file DataSheet2.PDF]

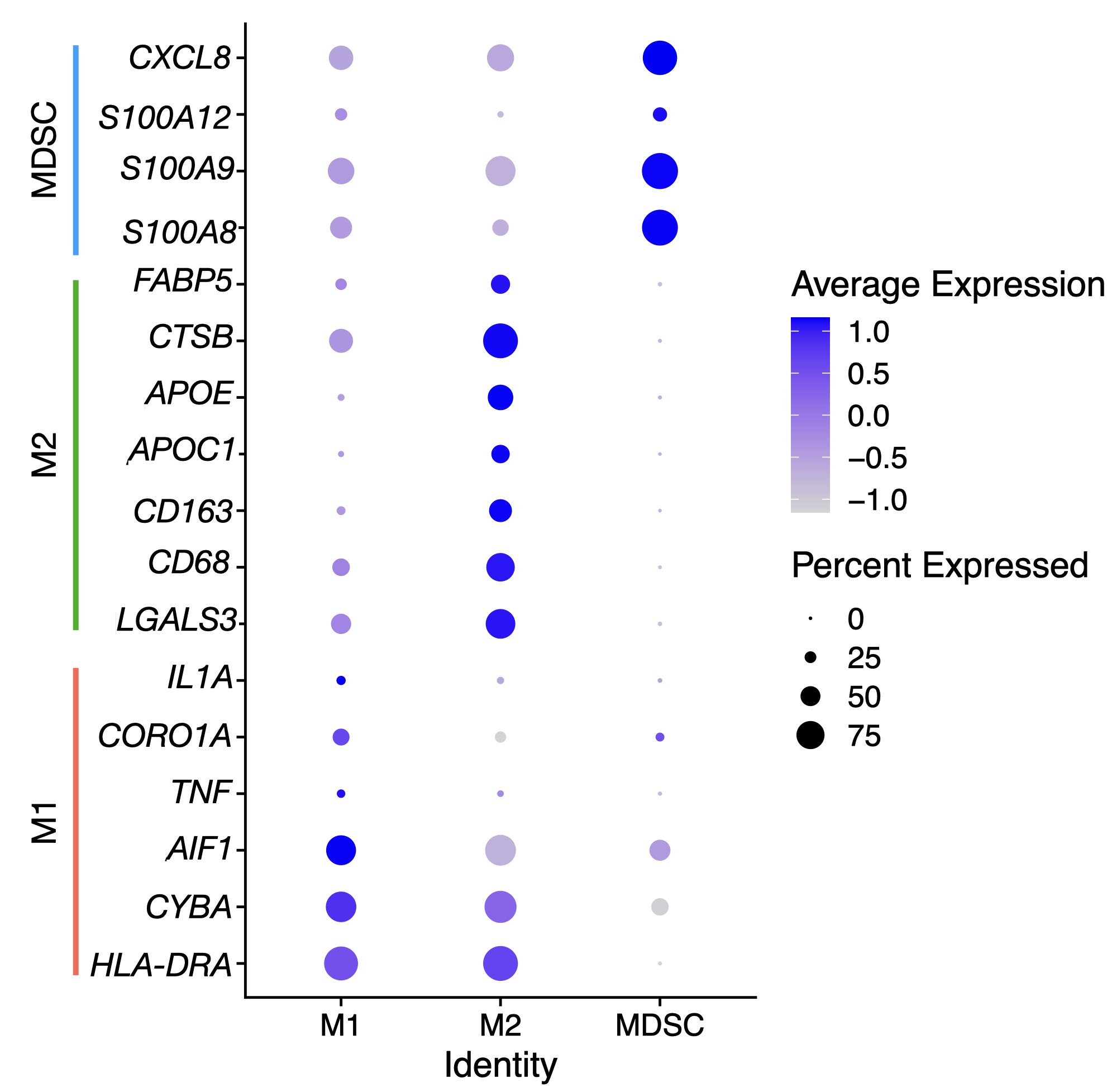

Supplement: Supplementary file 3 [file Image1.TIFF]

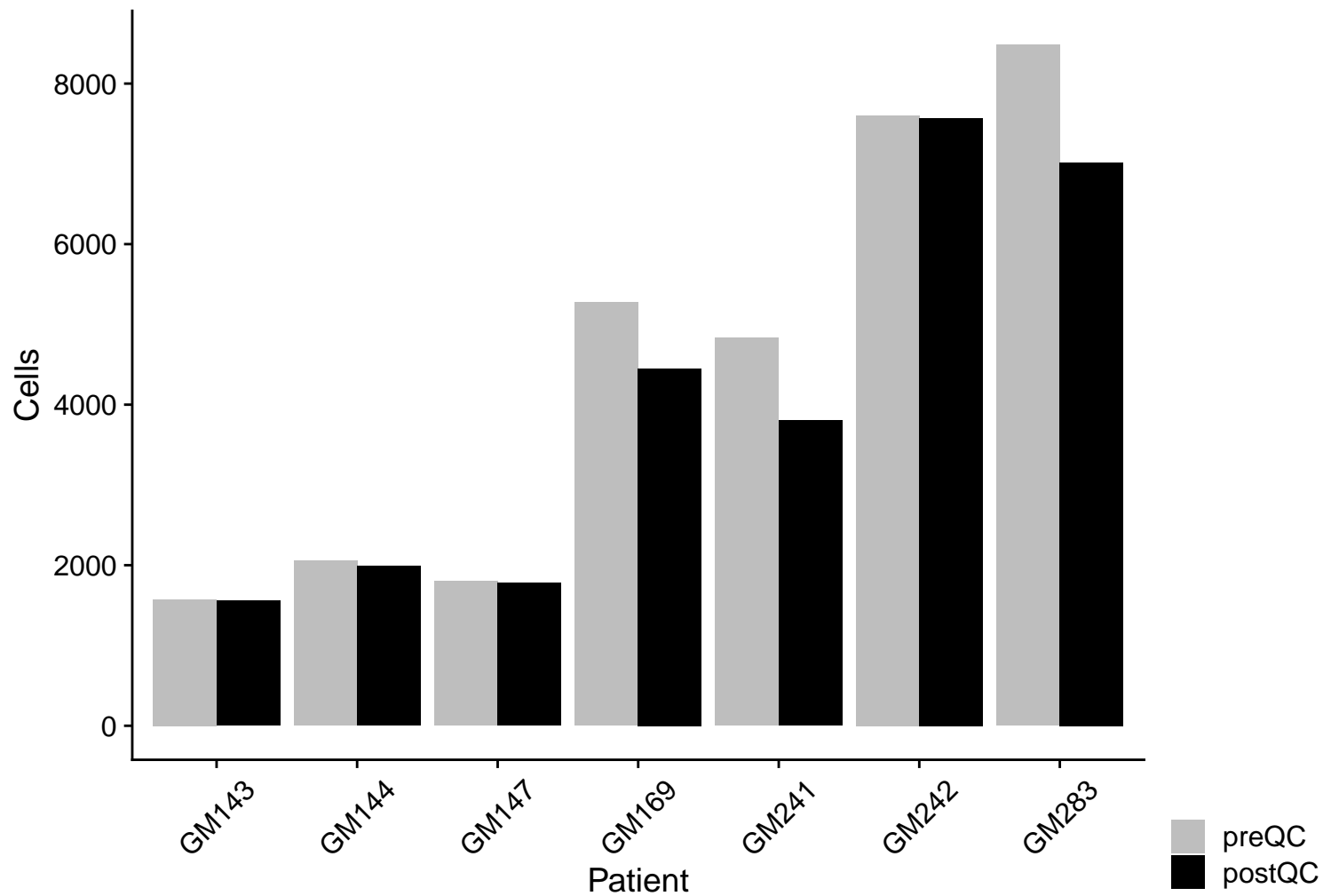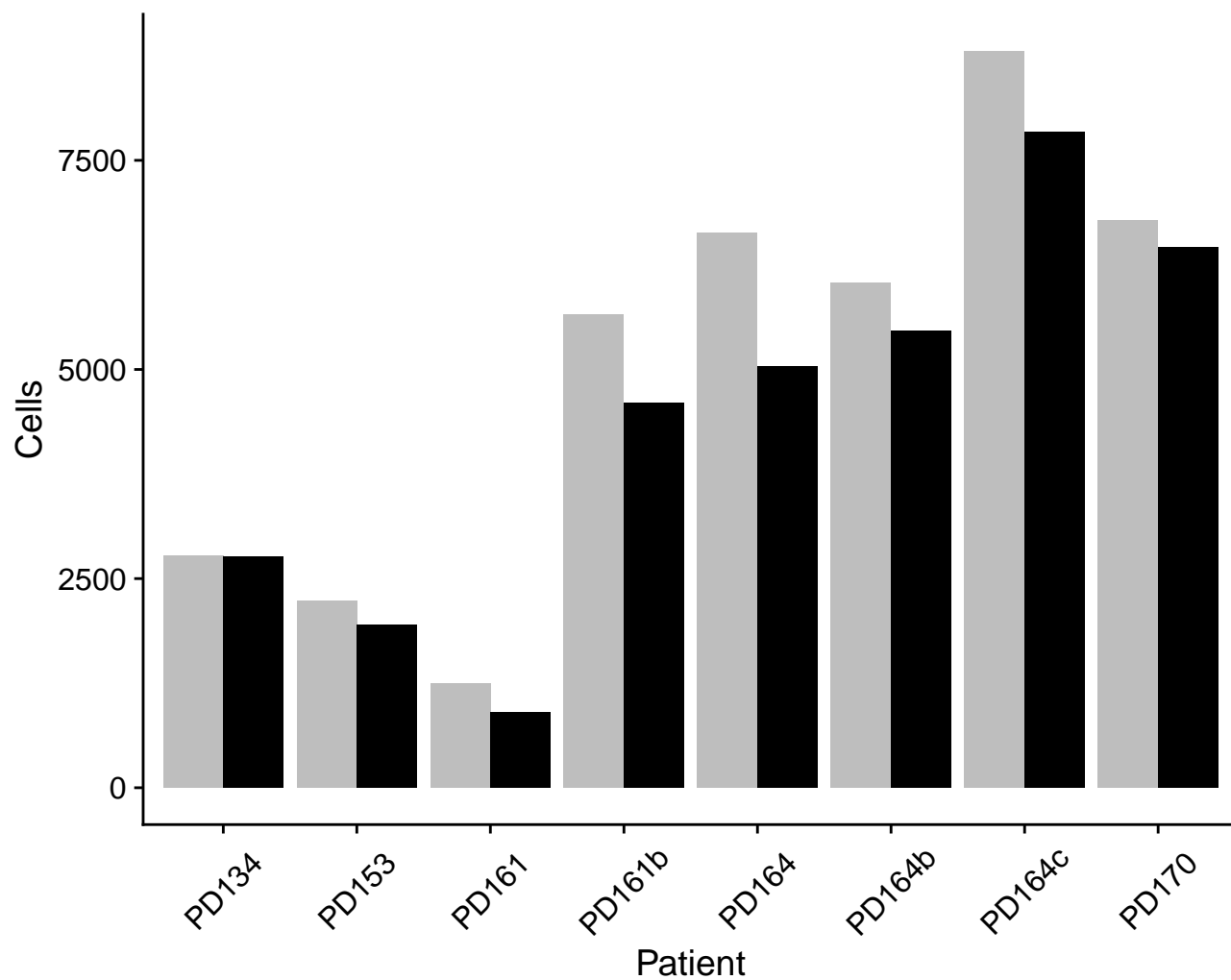

Supplement: Supplementary file 4 [file DataSheet3.PDF]

**nFeature\_RNA**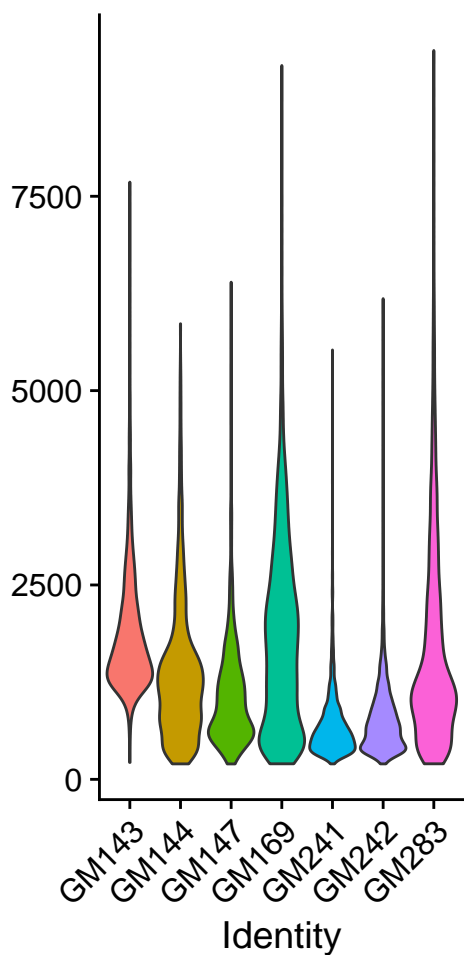**nCount\_RNA**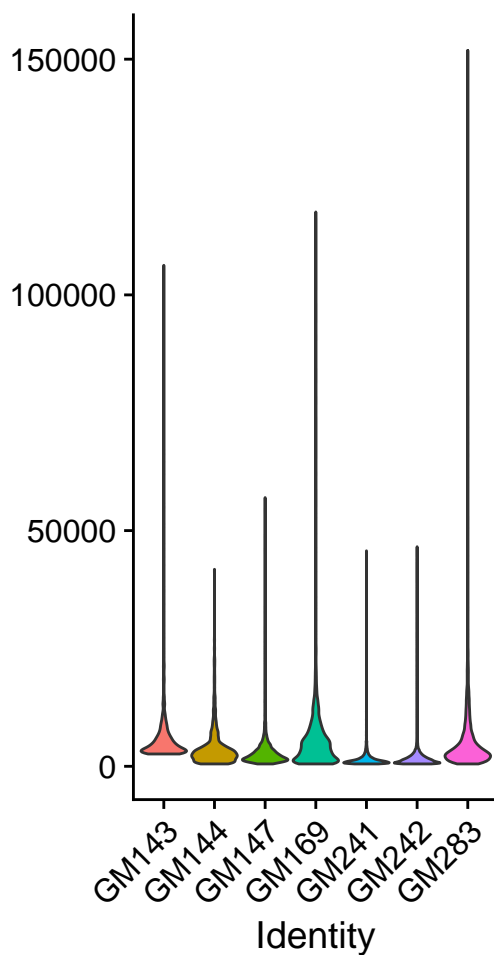**percent.mt**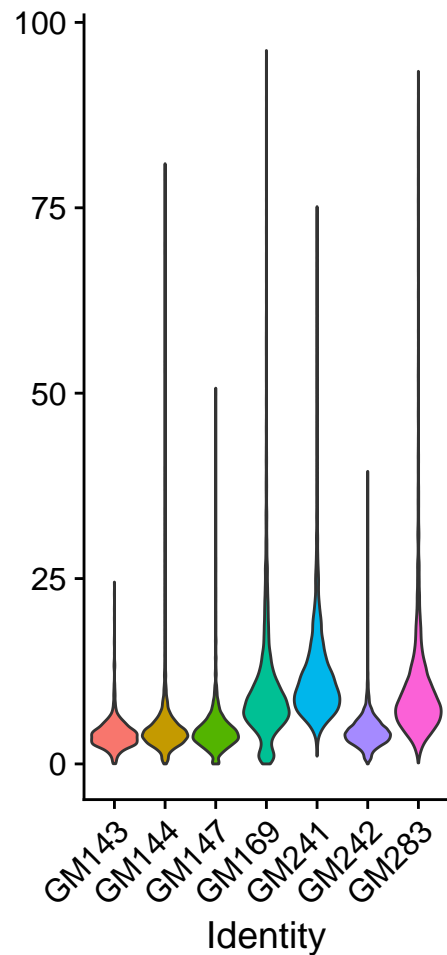**nFeature\_RNA**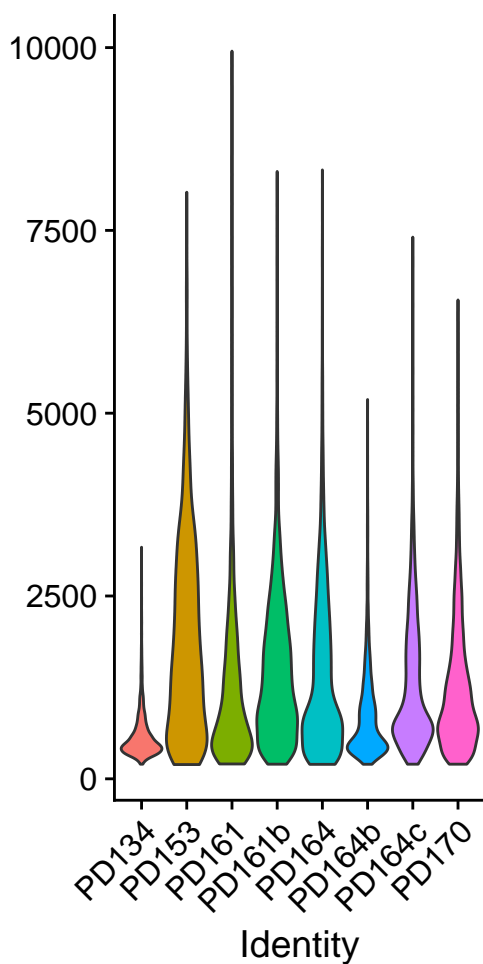**nCount\_RNA**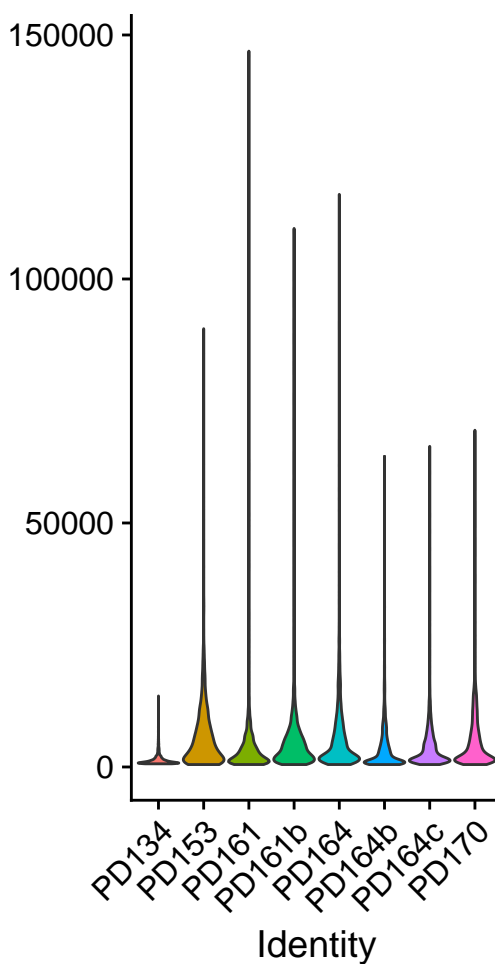**percent.mt**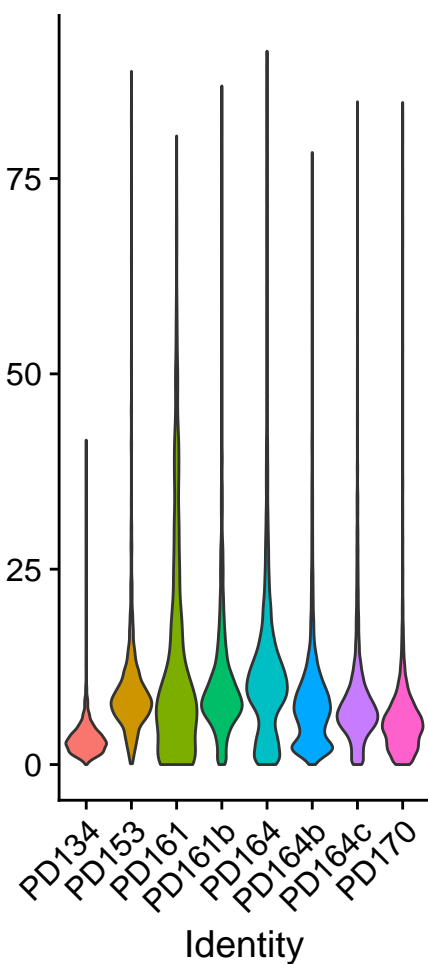

Supplement: Supplementary file 5 [file DataSheet1.PDF]
